# Protacs in oral cancer: degrading oncogenic drivers for next-generation therapy

**DOI:** 10.3389/froh.2026.1691816

**Published:** 2026-02-25

**Authors:** Pratibha Prasad, Manal Jamil Al Maslamani, Khuzin Dinislam, Syed Tasqeruddin, Anas Shamsi

**Affiliations:** 1 Basic Medical and Dental Sciences Department, College of Dentistry, Ajman University, Ajman, United Arab Emirates; 2 Center of Medical and Bio-allied Health Sciences Research, Ajman University, Ajman, United Arab Emirates; 3 Clinical Dental Sciences, College of Dentistry, Ajman University, Ajman, United Arab Emirates; 4 Department of General Chemistry, Bashkir State Medical University, Ufa, Russia; 5 Department of Pharmaceutical Chemistry, College of Pharmacy, King Khalid University, Abha, Saudi Arabia

**Keywords:** head and neck squamous cell carcinoma, precision oncology, protein-protein interaction inhibitors, proteolysis-targeting chimeras (PROTACs), signal transducer and activator of transcription 3 (STAT3), targeted protein degradation

## Abstract

Oral squamous cell carcinoma (OSCC) remains difficult to treat because of its intricate molecular profile, its limited responsiveness to conventional therapeutic approaches, and the challenge of targeting key oncogenic drivers with standard drugs. An emerging approach that addresses these limitations is the use of proteolysis-targeting chimeras (PROTACs), which shifts the focus from traditional inhibition of protein activity to the deliberate degradation of disease-associated proteins. PROTACs can eliminate oncogenic proteins like EGFR, STAT3, c-MYC, and anti-apoptotic regulators by hijacking the ubiquitin-proteasome system, many of which are essential for OSCC pathophysiology and are considered undruggable. This method provides a catalytic, sustained mechanism of action and overcomes the resistance arising from target overexpression, mutation, or signaling redundancy. Recent advances in PROTAC design, consisting of orally bioavailable degraders and tissue-directed delivery systems, highlight their translational capacity in epithelial tumors. PROTACs enable degradation of critical effectors involved in proliferation, immune evasion, and therapy resistance in OSCC. Hence, this review highlights how PROTAC technology addresses the current molecular targeting gaps in OSCC and outlines future directions for translating targeted protein degradation into clinical therapy.

## Introduction

Oral squamous cell carcinoma (OSCC) accounts for most oral malignancies worldwide, with over 377,000 new cases and 177,000 deaths reported in 2021 (1, 2). The disease burden is highest in South and Southeast Asia, particularly India, China, Pakistan, Bangladesh, and Indonesia, where tobacco, betel quid, and alcohol use are extensive (2–4). In India, OSCC is the leading cancer among men, contributing to nearly 30% of cases, with rising incidence and mortality across Southeast Asia due to limited prevention and late detection. Southeast Asia continues to report increasing trends in both incidence and mortality, indicating inadequate prevention and late diagnosis (1, 5–7). OSCC shows a high mortality-to-incidence ratio due to aggressive growth, delayed diagnosis, poor access to treatment, and therapy resistance (3, 8). Advances in surgery, radiotherapy, and chemotherapy have not improved global 5-year survival, which remains around 50%–60%. Standard regimens targeting EGFR or VEGFR show limited benefit and frequent relapse (6, 8–11). Conventional small-molecule inhibitors rely on occupancy-based inhibition, which often fails in the presence of overexpressed or mutant oncoproteins. Many cancer-promoting proteins, such as transcription factors (e.g., c-MYC, STAT3), have traditionally been considered “undruggable” due to a lack of defined binding pockets (12–14).

### PROTAC

Protein degradation technologies such as proteolysis targeting chimeras (PROTACs) offer an alternative approach by eliminating target proteins rather than inhibiting their activity (15–17). PROTACs are bifunctional molecules comprising three parts: a ligand or “warhead” that binds the protein of interest, a linker, and a ligand that recruits an E3 ubiquitin ligase such as von Hippel–Lindau (VHL), cereblon (CRBN), or MDM2 (16–18). The PROTAC bridges the target protein and E3 ligase, promoting ubiquitination and subsequent proteasomal degradation (19–21). This catalytic mechanism allows PROTACs to function at sub-stoichiometric levels and degrade even non-enzymatic and transcriptional regulators, including proteins with no active site for small molecule binding (16, 22). PROTACs eliminate the protein from the cellular environment, offering a potential solution to resistance caused by target overexpression or mutation when compared to traditional inhibitors (15, 16).

### PROTAC application in OSCC

OSCC frequently involves overactivation of EGFR, persistent STAT3 signaling, overexpression of anti-apoptotic proteins such as BCL-2 and MCL-1, and upregulation of cell cycle regulators such as c-MYC and CDK6 (10, 12, 14). EGFR inhibitors such as erlotinib and cetuximab have shown limited success in clinical trials due to compensatory activation of STAT3 or PI3K pathways (9, 10). Direct inhibition of STAT3 has been difficult using conventional inhibitors, but PROTAC molecules have demonstrated potent degradation of STAT3 in preclinical studies, overcoming certain drawbacks of occupancy-based inhibition (9, 13) Similarly, MYC, a master regulator of proliferation and metabolism, has long been considered undruggable due to its intrinsically disordered structure. Targeting MYC with PROTACs results in significant downregulation of downstream oncogenic signals, leading to apoptosis and tumor regression in experimental cancer models (14, 22). MCL-1, an anti-apoptotic member of the BCL-2 family often overexpressed in OSCC, also represents an actionable target for PROTAC-mediated degradation. This strategy may sensitize tumors to chemotherapeutic agents and enhance apoptotic responses (16, 21).

In cancer biology, PROTAC targets intersect directly with core hallmarks of cancer as defined by Hanahan and Weinberg (23). For instance, EGFR is a key driver of the hallmark *sustaining proliferative signaling*, a process that is frequently dysregulated in OSCC through receptor overexpression and constitutive activation. Additionally, downstream of EGFR, STAT3 signalling leads to multiple oncogenic processes, thereby resulting in enhanced proliferation, apoptosis resistance and inflammation-associated tumour progression. pEGFR and pSTAT3, which are phosphorylated forms of these proteins, are the biologically active oncogenic states that can be associated with OSCC pathogenesis most closely. Thus, PROTAC-based strategies that selectively target the degradation of these active states offer a mechanistically rational approach in oral cancer therapeutics.

The advantages of PROTACs sub-stoichiometric action, potential to target undruggable proteins, and reduced risk of acquired resistance make them particularly attractive for OSCC therapy (17, 20, 22). Integrating these compounds into therapeutic strategies could help overcome the current stagnation in OSCC treatment and could potentially lead to improved patient outcomes (24, 25).

This review brings together current knowledge on PROTAC design, key molecular targets relevant to OSCC, and the therapeutic potential of these agents to help guide future research toward more effective, mechanism-driven therapies for oral cancer.

## Molecular targets in oral cancer

**Figure 1:**
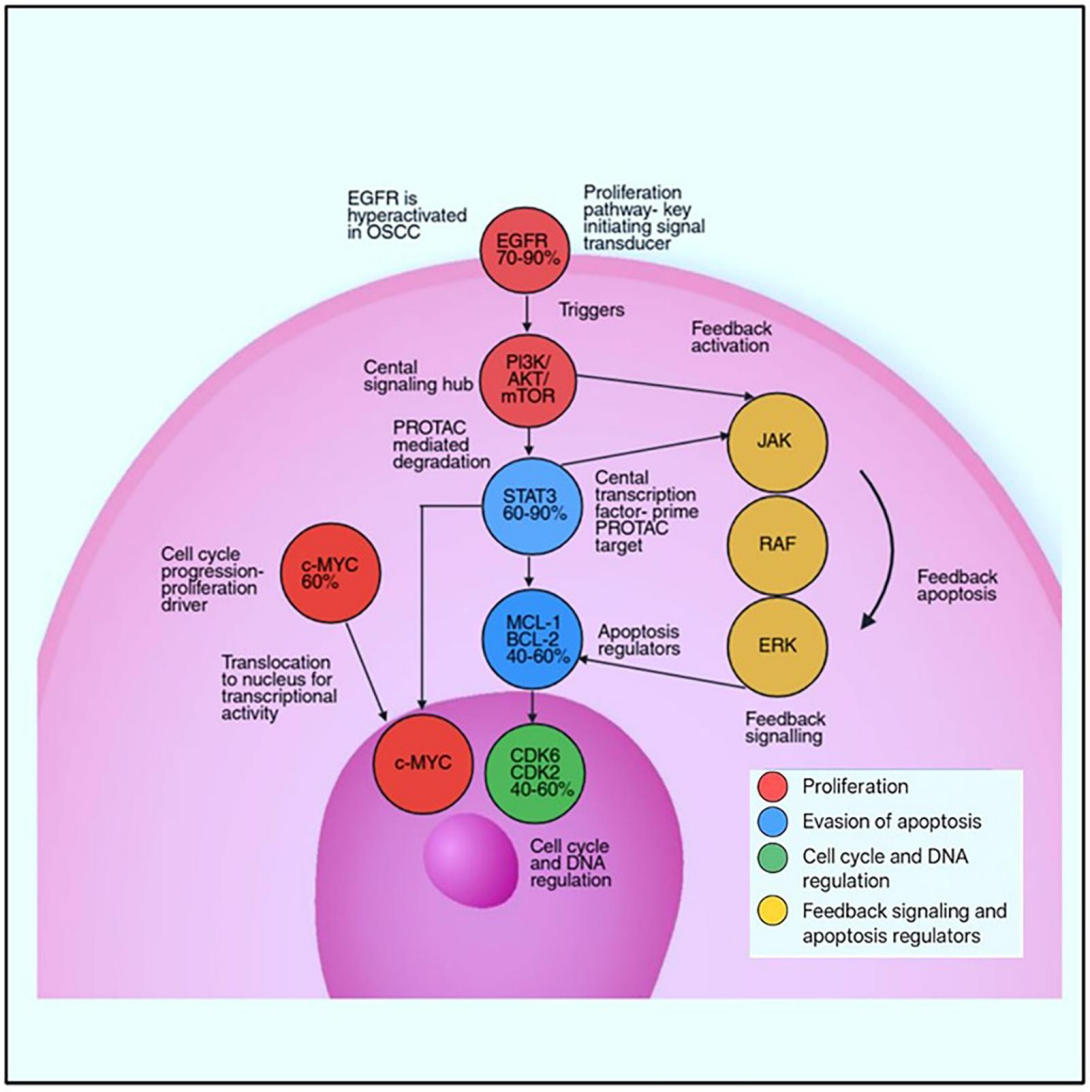
Dysregulated oncogenic signaling network in OSCC and points of PROTAC intervention. The illustrations show dysregulated oncogenic pathways in OSCC and highlight the therapeutic intervention points using PROTACs. The figure maps signal propagation from membrane-bound receptors to cytoplasmic and nuclear effectors, categorizing molecular nodes by function and illustrating feedback loops. Created in BioRender. Shamsi, M. (2025) https://BioRender.com/rcrm23q.

OSCC progression is driven by several key molecular pathways, including the EGFR family, STAT3, c-MYC, various cell-cycle regulators, and anti-apoptotic proteins such as BCL-2 and MCL-1. These factors not only support tumor growth but also exhibit resistance to standard therapeutic approaches, highlighting the need for alternative strategies like targeted protein degradation. The dysregulated oncogenic signaling network in OSCC and potential points of PROTAC intervention are illustrated in Figure 1.

### EGFR family

EGFR is overexpressed in 70%–90% of OSCC cases and plays a critical role in proliferation, angiogenesis, and cell survival through activation of the PI3K/AKT and MAPK signaling pathways (26). Cetuximab, a monoclonal antibody targeting the extracellular domain of EGFR, shows modest clinical efficacy. Monotherapy with cetuximab achieves an objective response rate of only 13%, with disease stabilization in approximately 46% of cases and a median overall survival of 5.8 months, in recurrent or metastatic OSCC (26). When combined with platinum and 5-fluorouracil in the EXTREME regimen, the overall response improves to 36%, with a median survival of 10.1 months compared to 7.4 months with chemotherapy alone (9). Small-molecule EGFR inhibitors such as erlotinib also show limited benefit in OSCC, with clinical trials reporting response rates as low as 4.3% (26). Resistance frequently develops through the activation of compensatory signaling cascades, particularly STAT3 and PI3K/AKT pathways, which sustain tumor growth independently of EGFR signaling (9, 27). Mutations in the EGFR kinase domain and downstream effectors contribute further to therapeutic failure. Complete degradation of EGFR and its dimerization partners via PROTACs may overcome these limitations.

### STAT3 and other transcription factors

Direct targeting of STAT3 with conventional inhibitors runs into several issues. This protein lacks a clearly defined ligand-binding site, and dimerization or phosphorylation-dependent activation frequently requires multiple interaction surfaces. Standard inhibitors often affect other signalling molecules, resulting in unintended off-target toxicities (27). Signal reactivation often occurs when STAT3 activity returns through feedback mechanisms involving SRC family kinases or JAK kinases, leading to only transient suppression rather than sustained inhibition (28).

### c-MYC and cell cycle regulators

c-MYC regulates genes involved in cell cycle progression, ribosomal biogenesis, and metabolic adaptation. Overexpression of c-MYC occurs in approximately 60% of OSCC tumors and correlates with aggressive proliferation, genomic instability, and therapeutic resistance (14, 22). Structurally, c-MYC lacks defined ligand-binding pockets and functions through extensive protein–protein and protein–DNA interactions. Inhibitors aimed at disrupting the c-MYC–MAX heterodimer or targeting downstream signaling have shown suboptimal clinical responses (22). Although c-Myc is a well-established oncogene with a critical role in transcription and cell proliferation, current evidence does not advocate its use as an independent prognostic marker in OSCC (29). Thus, its relevance in oral cancer is therefore primarily biological rather than prognostic.

Cell cycle dysregulation is a hallmark of OSCC, with cyclin-dependent kinases (CDKs) playing a central role. CDK6 and CDK2 are frequently overexpressed and contribute to unchecked progression through G1/S and G2/M checkpoints (30). Cell cycle dysregulation should be considered as a downstream consequence of core cancer hallmarks, viz., sustaining proliferative signalling, evasion of growth suppressors, and cell death resistance and not a solitary hallmark (23). Pharmacologic inhibition of CDK4/6 has demonstrated moderate antitumor efficacy in preclinical OSCC models. However, compensatory activation of alternative CDK complexes, particularly cyclin E–CDK2 and c-MYC–CDK axis, limits durable response (14).

Previous studies have reported a synergistic interaction between c-Myc and Bcl-2 in suppressing apoptosis, thereby promoting abnormal proliferation. However, in OSCC, the actual contribution of this interaction leading to tumor progression, aggressiveness, and prognosis remains poorly defined.

### Anti-apoptotic proteins (BCL-2, MCL-1)

Anti-apoptotic members, particularly BCL-2 and MCL-1, are overexpressed in nearly 40%–60% of OSCC cases (30). Elevated expression of BCL-2 and MCL-1 correlates with therapy resistance, reduced apoptosis following chemotherapy, and adverse overall outcomes. BH3 mimetics, which inhibit BCL2, have shown efficacy in haematological cancers. In OSCC and other solid tumours, however, their therapeutic impact is significantly reduced. One key reason is the compensatory regulation of MCL-1, which restores anti-apoptotic function when BCL-2 is inhibited (27).

PROTACs offer a promising alternative. They remove rather than inhibit proteins, and overcome resistance seen with traditional therapies (13, 16). Their catalytic action allows efficient, sustained suppression, making PROTACs a feasible therapeutic approach for overcoming OSCC drug resistance. Key oncogenic drivers in OSCC and their corresponding PROTACs-based therapeutic strategies are summarized in Table 1.

**Table 1:** PROTAC-Based therapeutic targeting of Key oncogenic drivers in OSCC.

Molecular target/oncogenic driver	Dysregulation in OSCC	Oncogenic function	Limitations of current therapeutics	PROTAC example (name)	Cancer type	E3 ligase	Therapeutic outcome	Reference(s)
STAT3	60%–90% overexpression	Regulates proliferation, survival, immune evasion	No defined binding pocket, feedback activation via JAK/Src	SD-36	Oral SCC, epithelial cancers	CRBN	Induced STAT3 degradation; tumor growth inhibition	(12, 13)
EGFR	70%–90% overexpression (1, 53)	Proliferation via MAPK/PI3K signaling (53, 71)	Resistance via mutations and pathway bypass; transient inhibition (23, 53)	—	OSCC (preclinical)	—	EGFR-STAT3 axis inhibition; resistance reversal	(9, 10, 12, 30)
BRD4	Overexpressed in HNSCC (25)	Transcriptional activation, c-MYC upregulation (20, 25)	Lack of catalytic site; redundancy in transcriptional cofactors (11)	ARV-825, dBET6	Head & Neck SCC, OSCC	CRBN	MYC suppression, antiproliferative effects	(14, 28, 34)
c-MYC	∼60% overexpression (20, 40)	Drives proliferation, metabolic shift (20)	Undruggable dimerization interface; off-target effects (25, 40)	—(via BRD4 targeting)	OSCC	CRBN (via BRD4)	Indirect MYC suppression through BRD4 degradation	(14, 28)
BCL-xL	Elevated in OSCC (55)	Blocks apoptosis, chemoresistance (55)	Resistance via MCL-1 redundancy; toxicity (25, 55)	DT2216	Head & neck, solid tumors	VHL	Selective tumor degradation; low toxicity	(72, 74)
MCL-1	Highly expressed, linked to poor prognosis (55)	Resistance to apoptosis	Short half-life, structural flexibility, cardiac toxicity with inhibitors (55)	—	OSCC (preclinical rationale)	—	Potential target for degradation-based elimination	(12, 28)
Estrogen Receptor (ER)	Model for theranostic PROTACs	Transcription regulation	Used in design of imaging-compatible degraders (47, 73)	ARV-471	Breast cancer	VHL	ER*α* degradation; PET/fluorescence theranostics	(49, 58)
CDK9	Dysregulated cell cycle control (71)	Controls transcription elongation and cell cycle progression (20)	High toxicity; partial suppression with inhibitors (20, 34)	THAL-SNS-032	SCC including oral	CRBN	Induced cell cycle arrest	(14, 18, 28)
HPK1	Immunoregulatory node	Suppresses T-cell activation	No OSCC-specific data; high relevance in imm					(56)

OSCC, oral squamous cell carcinoma; SCC, squamous cell carcinoma; HNSCC, head and neck squamous cell carcinoma; EGFR, epidermal growth factor receptor; STAT3, signal transducer and activator of transcription 3; BRD4, bromodomain-containing protein 4; MYC, myelocytomatosis oncogene; BCL-xL, B-cell lymphoma-extra large; MCL-1, myeloid cell leukemia 1; ER, estrogen receptor; AR, androgen receptor; CDK9, cyclin-dependent kinase 9; HPK1, hematopoietic progenitor kinase 1; BET, bromodomain and extra-terminal motif; EZH2, enhancer of zeste homolog 2; VEGFR-2, vascular endothelial growth factor receptor 2; MDM2, mouse double minute 2 homolog; CRBN, cereblon; VHL, von hippel-lindau; PET, positron emission tomography.

## Mechanism of PROTACS

PROTACs are heterobifunctional molecules that enable selective degradation of intracellular proteins via the ubiquitin–proteasome pathway and consist of 3 main domains: a ligand that binds the protein of interest (POI), a linker, and a recruiter moiety that binds an E3 ubiquitin ligase (15–17). Once inside the cell, PROTACs induce proximity between the target protein and an E3 ligase, facilitating ubiquitination of the target. The tagged protein is subsequently recognized by the 26S proteasome, which degrades it completely, leading to full elimination rather than mere inhibition of its activity (16, 19, 21).

### Structure of PROTAC

Each PROTAC molecule contains a warhead, which may be derived from non-inhibitors, peptides, or natural compounds with high affinity for the target (17). The linker connects the warhead to the E3 ligase ligand and is critical in maintaining flexibility, spatial arrangement, and stability of the ternary complex. The E3 ligase recruiter binds an endogenous E3 ubiquitin ligase, such as von Hippel–Lindau (VHL), cereblon (CRBN), or murine double minute 2 (MDM2) (16–18). The ternary complex formed by the PROTAC, the POI, and the E3 ligase catalyzes polyubiquitination of the target protein. This enables the proteasome to recognize and degrade the protein. Unlike occupancy-based inhibitors that temporarily block a functional site, PROTACs induce catalytic degradation, allowing sub-stoichiometric quantities to achieve sustained suppression (16, 20).

### Commonly recruited E3 ligases and their relevance in oral cancer

More than 600 E3 ligases exist in human cells, but only a few have been effectively applied in PROTAC development because of ligand availability and biochemical compatibility. The most widely used are cereblon (CRBN), which functions as part of the CRL4–CRBN E3 ligase complex and is recruited by thalidomide derivatives such as pomalidomide and lenalidomide (16, 18); von Hippel–Lindau (VHL), a component of the CRL2–VHL complex that recognizes hydroxylated proline residues in hypoxia-inducible factors (HIFs) and whose ligands have been optimized for oral bioavailability, supporting their broad application in PROTACs targeting kinases, transcription factors, and oncogenic fusion proteins (17, 21); and MDM2, which regulates p53 and has been utilized in PROTAC design, particularly for dual-targeted cancer therapies in tumors retaining wild-type p53 (17). CRBN and VHL are expressed in epithelial tissues, including head and neck mucosa, indicating their accessibility for PROTAC-mediated degradation in oral cancers such as OSCC (18, 21), but comprehensive transcriptomic and proteomic profiling of oral epithelium is needed to determine the most effective ligase for OSCC-targeted PROTACs.

### Pharmacological advantages of PROTACs

PROTACs exhibit numerous advantages over conventional small-molecule inhibitors. Firstly, they do not require high-affinity binding to the functional site of the target protein. Even weak binding ligands can initiate degradation, provided they support effective ternary complex formation (16, 17). Secondly, PROTACs function catalytically, and hence they can degrade multiple copies of the POI per molecule, which reduces the need for high systemic concentrations and lowers off-target toxicity (17, 20). They can also target non-enzymatic and structurally disordered proteins, such as transcription factors (e.g., STAT3, c-MYC), which are typically considered “undruggable” due to the lack of deep hydrophobic pockets for inhibitor binding (13, 14, 22). This expands the druggable proteome significantly, providing new options to degrade chief oncogenic regulators in OSCC. Moreover, degradation of the entire protein prevents reactivation or compensation through post-translational modifications or alternative signaling loops, which is a common limitation with traditional inhibitors (12, 17). For example, EGFR-targeted therapies in OSCC often result in downstream activation of STAT3 or MAPK signaling, which maintains tumor cell survival. A PROTAC targeting EGFR would remove the entire receptor, reducing the likelihood of pathway escape (9, 10).

### Resistance and limitations

The effective application of PROTACs in solid tumours poses certain challenges. Their relatively high molecular weight and polarity often hinder cell membrane permeability and reduce oral bioavailability, limiting systemic delivery and bio-distribution (17, 31). Careful structural optimization of the linker and warhead components is necessary to achieve optimal pharmacokinetic and pharmacodynamic requirements (32, 33). In OSCC and other epithelial tumours, heterogeneity in E3 ligase expression may impact the efficacy of PROTAC-induced degradation (34). Identification of E3 ligases with stable and consistent expression in oral tissues is essential for successful translation (34). Some intracellular proteins may evade ubiquitination due to conformational masking or localization within subcellular compartments that restrict access (35). All targets are not equally susceptible to proteasomal degradation; hence, it is necessary to validate each PROTAC design in target tissues empirically (19, 35).

### Relevance in OSCC

The application of PROTACs in OSCC is especially important owing to the difficulty in targeting some of the main oncogenic modulators that use traditional approaches. For example, transcription factors such as STAT3 and c-MYC lack enzymatic activity and defined binding pockets, which make them inaccessible to most small molecule inhibitors (8, 12). BCL-2 family proteins, particularly MCL-1, have structural flexibility and short half-lives, limiting the efficacy of available inhibitors (12, 16). PROTACs that degrade these proteins directly may overcome such therapeutic resistance mechanisms in OSCC (22, 25). Moreover, PROTACs which target the EGFR family members may suppress not only the receptor itself but also downstream effectors. This potentially achieves a broader pathway suppression with less compensatory activation (9, 36). This is particularly relevant in OSCC, where EGFR overexpression and cross-talk with STAT3 and PI3K/AKT pathways regulate proliferation and resistance (9, 30, 37). Initial data from preclinical studies suggest that PROTACs targeting BRD4, AR, or ER have achieved potent degradation and antitumor responses in various solid tumours (16, 28, 38, 39).

### Design considerations for oral cavity application

Local delivery systems, including mucoadhesive films, nanoparticles, or hydrogels, facilitate the sustained release of PROTACs directly within the oral cavity, evading systemic metabolism and enhancing bioavailability in mucosal tissues. Selecting ligands with high specificity for OSCC targets, using stable linkers resistant to enzymatic degradation, and employing ligases abundantly expressed in oral tissue may optimise degradation efficacy and minimise off-target effects. Formulations allowing controlled local exposure may improve the therapeutic index and reduce systemic adverse effects (12, 19).

## PROTACS in cancer therapy

Head and neck cancers remain largely unexplored in PROTAC research. A recent study highlighted a STAT3-targeting PROTAC that degraded STAT3 and reduced downstream signalling in head and neck squamous carcinoma cells, thereby sensitising them to EGFR-targeted therapy (13, 27, 40). OSCC may particularly benefit from PROTACs that target key oncogenic drivers. EGFR-targeting PROTACs can eliminate the entire receptor protein, avoiding downstream reactivation via STAT3 or PI3K/AKT pathways (9, 26, 27).

PROTACs are effective in preclinical models of solid tumors and have demonstrated the degradation of kinase, transcription factor, and epigenetic targets. A VHL-recruiting PROTAC targeting BRD4 produced durable tumor regression in xenograft models and required doses over 20-fold lower than the parent inhibitor (17, 21, 38). A cereblon (CRBN)-based PROTAC selectively degraded ER*α* in breast cancer cells with nanomolar DC₅₀ values and overcame resistance to tamoxifen (41). Multiple orally bioavailable PROTACs have entered early clinical trials, including ARV-471 targeting the estrogen receptor and ARV-110 targeting mutant AR in prostate cancer (42, 43). Some studies have introduced new orally active PROTACs with improved pharmacokinetic profiles. A next-generation compound showed strong oral absorption and durable target engagement *in vivo*. These advances are appropriate with the increasing global impact of oral cancer and the demand for better treatment options (17, 21, 38). PROTACs targeting transcription factors have also shown promise. STAT3-specific degraders significantly reduced tumor growth in epithelial cancer models at sub-micromolar concentrations (13, 44). Preclinical studies demonstrated that degradation of c-MYC via PROTAC reduced cellular proliferation and induced apoptosis in aggressive solid tumor cell lines (14, 22, 45). PROTACs targeting BCL-2 and MCL-1 demonstrated synergistic effects with chemotherapy by removing key resistance-associated proteins (12). Clinical success of PROTACs in OSCC depends on translating strong laboratory data into human studies. Oral PROTACs like ARV-471 and ARV-110 have shown safe and measurable activity in early breast and prostate cancer trials (42, 43).

**Figure 2:**
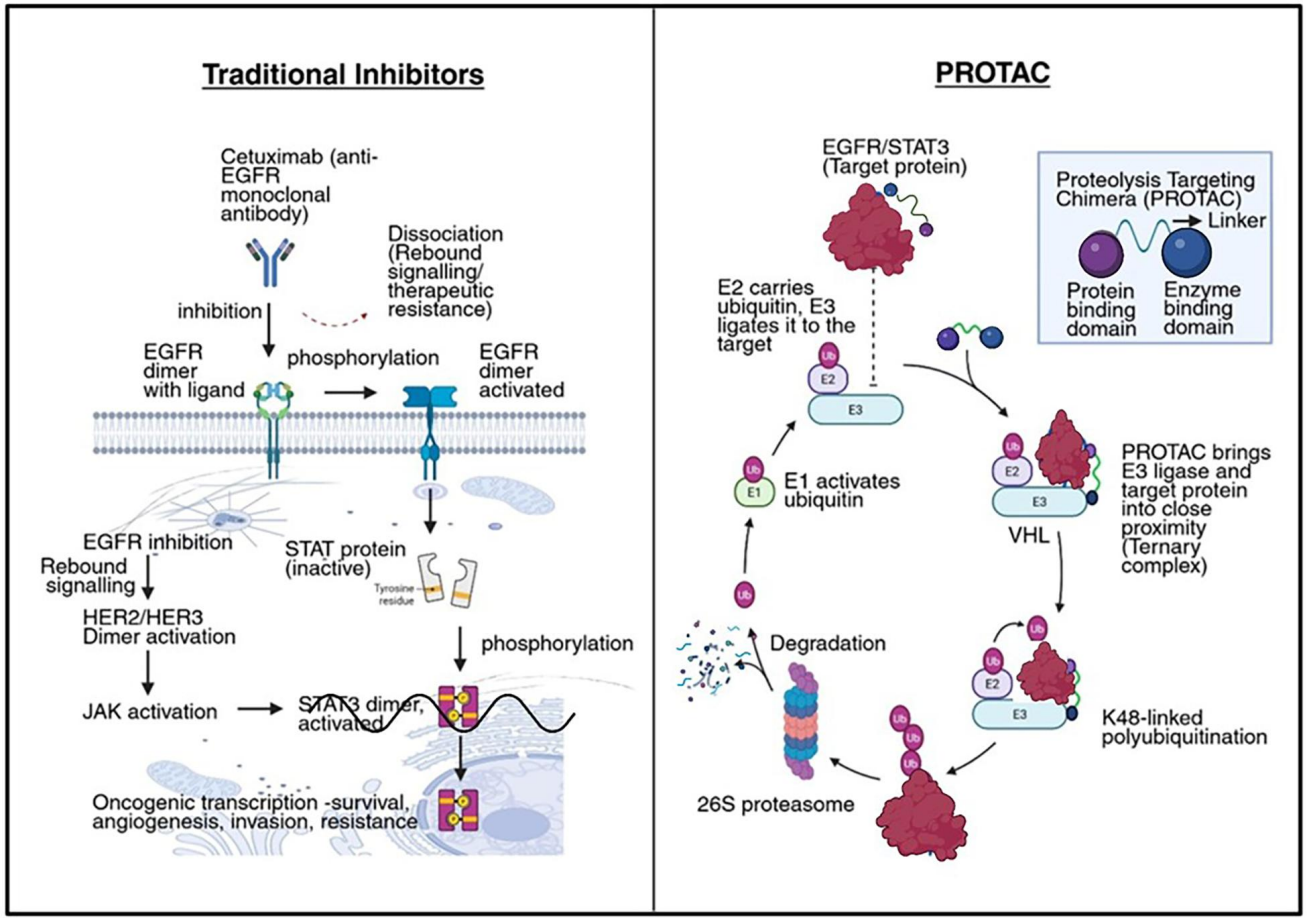
Mechanism of action of PROTACs in oncoprotein degradation in OSCC. Schematic illustration comparing traditional small-molecule inhibition with PROTAC-mediated targeted protein degradation. In the left panel, reversible binding of small molecules to oncoproteins like EGFR or STAT3 results in partial and temporary inhibition, often leading to signaling rebound and therapeutic resistance. The right panel depicts a bifunctional PROTAC molecule simultaneously binding the target protein and an E3 ligase (e.g., VHL), inducing K48-linked polyubiquitination and subsequent degradation via the 26S proteasome. The PROTAC is recycled, allowing catalytic degradation of multiple target proteins, including undruggable transcription factors, highlighting its therapeutic promise in OSCC. Created in BioRender. Shamsi, M. (2025) https://BioRender.com/2ctrlo1.

Human papillomavirus (HPV)-positive OSCC typically exhibits wild-type p53, low EGFR expression, and reliance on viral oncoproteins E6/E7. PROTACs directed against viral oncoproteins E6 and E7 could restore tumor suppressor functions such as p53 and Rb. MDM2-based PROTACs in HPV-positive cells could further enhance p53 stability through dual mechanisms (21, 46). Figure 2 illustrates the mechanistic difference between reversible inhibitors and PROTAC-mediated degradation. It shows how small molecules transiently block EGFR or STAT3, allowing rebound signaling. PROTAC binding of both target and E3 ligase (e.g., VHL) is demonstrated, which leads to K48-linked polyubiquitination and degradation by the 26S proteasome. PROTAC molecules are recycled, enabling catalytic action and sustained depletion of oncoproteins, including those traditionally considered “undruggable.”

Emerging oral-specific delivery platforms include nanoparticle-encapsulated PROTACs and buccal films engineered for sustained release in the oral cavity. These approaches may overcome challenges related to molecular size, poor membrane permeability, and salivary clearance (42, 47). PROTACs conjugated with radiotracers or fluorescent probes could provide real-time measurements of degradation and drug accumulation in tumor tissues (48, 49).

PROTACs have a high molecular weight, ranging between 800 and 1,100 Da, and increased polarity due to bifunctional design, which reduces membrane permeability and oral bioavailability compared to traditional small molecules (50, 51).

Resistance to PROTACs has emerged through several mechanisms, including reduced E3 ligase expression, mutations in E3 recruitment domains, or alterations in target protein ubiquitination sites (52). Loss-of-function mutations in CRBN and VHL have been observed in preclinical models and clinical samples, reducing degrader efficiency (53). Proteasomal inhibition or saturation, either by therapeutic agents or tumor-associated stress, may reduce degradation efficiency and promote drug persistence in intracellular compartments (53, 54).

Designing OSCC-specific PROTACs with proper delivery and combination strategies is important for improving the results in a cancer type that has resisted advances in pharmacologic therapy. A mechanistic and clinical comparison of current therapeutic modalities targeting oncogenic drivers in OSCC is summarized in Table 2. Identifying E3 ligases with high and consistent expression in oral tumors, but minimal in adjacent healthy tissue, remains a key step in OSCC-specific PROTAC development. Recent approaches aim to engineer artificial E3 ligase systems or screen for tissue-enriched ligases using proteogenomic data, but these strategies remain in early phases and require validation (54, 55). Thus, these molecular constraints guide the study of diagnostic and theranostic applications of PROTACs in oral cancer.

**Table 2:** Mechanistic and clinical comparison of therapeutic modalities targeting oncogenic drivers in OSCC.

Therapeutic strategy	Primary mechanism of action	Clinical application challenges in OSCC	Ability to target “undruggable” proteins	Resistance mechanisms	Pharmacokinetic considerations	Delivery systems	Advantages of PROTACs over This modality	References
Conventional chemotherapy (e.g., Cisplatin, 5-FU)	DNA crosslinking, inhibition of DNA synthesis	Non-specific toxicity, poor survival outcomes, chemoresistance	No	DNA repair upregulation, drug efflux (e.g., MDR1), apoptosis evasion	Short half-life, systemic toxicity	IV injection	Selective degradation of oncogenic drivers with reduced systemic toxicity	(68)
Targeted therapy (EGFR inhibitors—Cetuximab, Erlotinib)	Blockade of receptor tyrosine kinases and downstream pathways (e.g., MAPK/PI3 K)	Limited to patients with EGFR overexpression; limited response in OSCC	No (receptor-dependent)	EGFR mutations, STAT3 bypass, feedback loops	Variable bioavailability	IV/oral	PROTACs can degrade EGFR or bypass resistance via STAT3 degradation	(9, 10)
Immune checkpoint inhibitors (PD-1/PD-L1, CTLA-4)	Reactivation of cytotoxic T-cell response	Tumor microenvironment heterogeneity, low response in OSCC	No	Immune evasion, PD-L1 upregulation, Treg dominance	Long half-life, immune-related adverse events	IV/Immunoliposomes	PROTACs can reprogram immune environment via degradation of immune evasion mediators (e.g., HPK1)	(75)
RNA interference (siRNA, shRNA)	mRNA knockdown via RISC complex	Delivery barriers, instability *in vivo*, transient effects	Partial (if mRNA is targetable)	RNAi silencing inefficiency, off-target effects	Rapid degradation, requires encapsulation	Liposomes, nanoparticles	PROTACs offer persistent degradation at the protein level, not mRNA	(34)
Monoclonal antibodies (e.g., anti-EGFR)	Target extracellular domains of membrane receptors	Ineffective against intracellular or truncated receptors; immunogenicity	No	Receptor mutation, ligand-independent activation	High molecular weight limits tissue penetration	IV only	PROTACs act intracellularly and degrade both wild-type and mutant proteins	(37)
Small-molecule inhibitors (SMIs)	Occupy active/binding sites of oncogenic kinases	Ineffective against scaffolding/non-enzymatic proteins	No	Point mutations in binding pocket, feedback activation	Orally available but prone to metabolic clearance	Oral	PROTACs work catalytically, degrading even scaffold or mutant proteins	(51, 76)
Epigenetic inhibitors (e.g., HDAC, BET inhibitors)	Disruption of chromatin remodeling and gene expression	Off-target transcriptional reprogramming, transient effects	Limited	Epigenetic compensation, pathway redundancy	Variable depending on class	Oral/injection	PROTACs can degrade entire complexes (e.g., BRD4, EZH2), inducing complete silencing	(28, 34)
PROTACs (proteolysis-targeting chimeras)	E3 ligase-mediated targeted degradation of intracellular proteins	Developmental stage in OSCC; bioavailability & tumor penetration optimization needed	Yes	Resistance via E3 ligase alteration, proteasome adaptation	Oral PROTACs under development; improved half-life and selectivity	Oral, nanoparticles, peptide conjugates	Simultaneous modulation of oncogenic, epigenetic, and immune pathways; sustained suppression	(25, 38, 45)

OSCC, oral squamous cell carcinoma; PROTAC, proteolysis-targeting chimera; STAT3, signal transducer and activator of transcription 3; BRD4, bromodomain-containing protein 4; CDK9, cyclin-dependent kinase 9; BCL-xL, B-cell lymphoma-extra large; EGFR, epidermal growth factor receptor; ICI, immune checkpoint inhibitor; RNAi, RNA interference; siRNA, small interfering RNA; LNP, lipid nanoparticle; HNSCC, head and neck squamous cell carcinoma.

### Diagnostic and theranostic applications of PROTACs in OSCC

**Figure 3:**
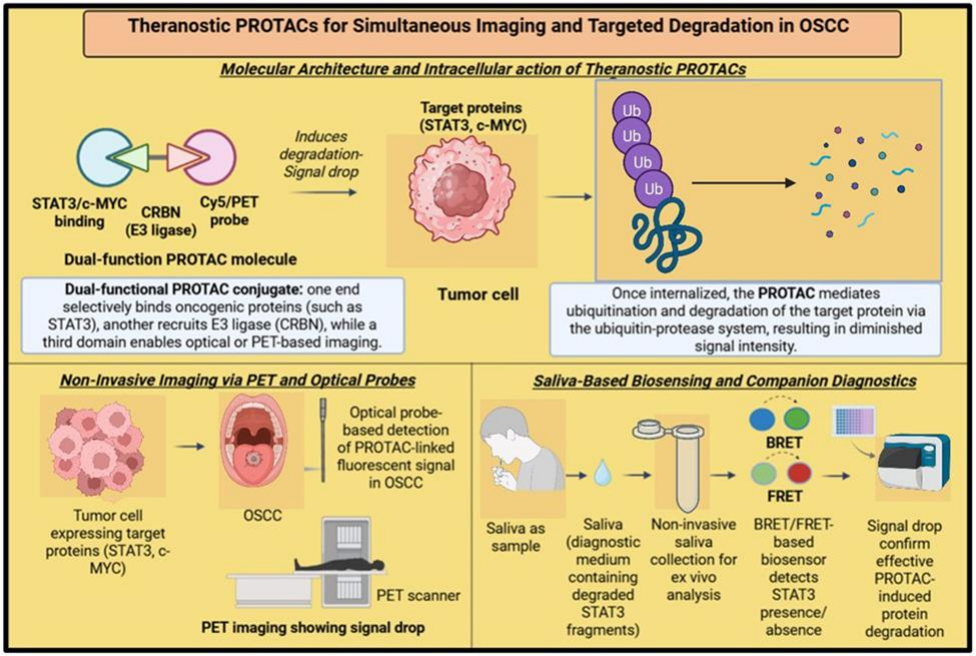
Theranostics PROTACs for simultaneous imaging and degradation in OSCC. Schematic illustration showcases theranostic PROTACs designed for simultaneous imaging and targeted degradation in OSCC. It explains dual-function PROTAC architecture, PET/optical probe imaging, and saliva-based BRET/FRET biosensing. The image emphasizes non-invasive diagnostics, oncoprotein degradation (e.g., STAT3, c-MYC), and real-time signal drop detection for precision therapy and companion diagnostics. Created in BioRender. Shamsi, M. (2025) https://BioRender.com/y2rt6bn.

Visualizing protein degradation in real-time provides an accurate approach for evaluating therapeutic response in OSCC. PROTACs conjugated with imaging tags have enabled dual-action molecules that degrade targets and simultaneously provide molecular imaging feedback (Figure 3). Fluorescence and positron emission tomography (PET) platforms have been successfully adapted to track intracellular protein degradation using PROTAC constructs. One study developed a NAMPT-targeting PROTAC conjugated with a coumarin-based fluorophore, allowing visualization of degradation kinetics and intracellular localization both *in vitro* and in xenograft models (56). Another construct targeting estrogen receptor alpha (ER*α*) was modified with BODIPY dye, enabling fluorescent imaging of ER*α* degradation in breast cancer models (49). Both approaches serve as models for future constructs applicable to OSCC targets such as STAT3, c-MYC, or EGFR, which are often overexpressed and difficult to quantify through static biopsy or immunohistochemistry (28, 40, 57). PET-based theranostic PROTACs have also shown translational promise. Environment-sensitive PROTACs were designed to emit PET signals upon degradation of target proteins, enabling non-invasive longitudinal monitoring of therapy response (49, 56, 58). These could be used in OSCC for visualizing target engagement in locoregional lesions or in monitoring second primaries, a significant concern in field cancerization. PROTACs designed for dual function not only degrade oncogenic proteins but also serve as chemical probes to assess molecular dependencies *in vivo* (35, 59). Targeted degradation of STAT3 or EGFR in OSCC xenografts has been associated with reduction in PET signal intensity and correlated tumor regression, supporting the use of such systems for confirming biological relevance and functional suppression (49, 56, 58).

PROTACs have also been integrated into biosensing platforms. Förster resonance energy transfer (FRET)- and bioluminescence resonance energy transfer (BRET)-based biosensors employing PROTAC motifs allow real-time detection of protein–protein interactions, target occupancy, and degradation susceptibility (34, 35). These systems are adaptable for OSCC-related targets such as BRD4, CDK6, or NF-κB, where validation of degradation is required before clinical translation (28, 34). Nanoparticle-mediated delivery of imaging-enabled PROTACs has been another successful innovation. Nanocarriers tagged with Cy5 or quantum dots have delivered PROTACs to tumors while allowing real-time visualization via near-infrared or fluorescence imaging (49, 58, 60). This strategy is particularly advantageous in OSCC where the oral cavity allows direct or localized application, limiting systemic exposure and allowing repeated assessment without cumulative radiation burden.

Theranostic PROTACs provide a platform that integrates targeted degradation with molecular imaging in OSCC. These agents permit non-invasive, real-time monitoring of oncoprotein elimination and correlate well with therapeutic efficacy. Integration with biosensors, nanoparticles, and fluorescence/PET imaging is feasible and allows continuous feedback during therapy (35, 49, 60). PROTACs offer a rational approach to enhancing both diagnosis and treatment in OSCC, especially for targets considered undruggable using traditional pharmacologic inhibitors (Figure 3).

## PROTACS as chemical biology probe in OSCC research

Because PROTACs act reversibly, they can serve as effective chemical probes; once treatment is withdrawn, protein levels and function typically recover, unlike in permanent genetic knockouts. As PROTAC libraries continue to expand to encompass additional protein families such as ubiquitin ligases and epigenetic regulators, their utility in OSCC research is steadily increasing. Degraders directed against chromatin readers, kinases, and transcriptional coactivators are already under investigation in various squamous cancers and can be adapted for OSCC-specific studies (39, 45, 55).

## Formulation and delivery systems for oral cancer targeting

The accessible nature of the oral cavity offers opportunities for localized approaches that minimize systemic exposure. Several research groups have explored mucoadhesive nanoparticle platforms that adhere to the oral mucosa and slow-release therapeutic agents (5, 36). Exosomes derived from epithelial cells or engineered carriers can cross mucosal barriers and selectively deliver therapeutic molecules to epithelial cancers. Liposomal delivery of large molecules, including peptides and oligonucleotides, has shown efficacy in head and neck models. PROTAC encapsulation in similar carriers is under development and may offer enhanced penetration and controlled degradation (24, 38, 50).

**Figure 4:**
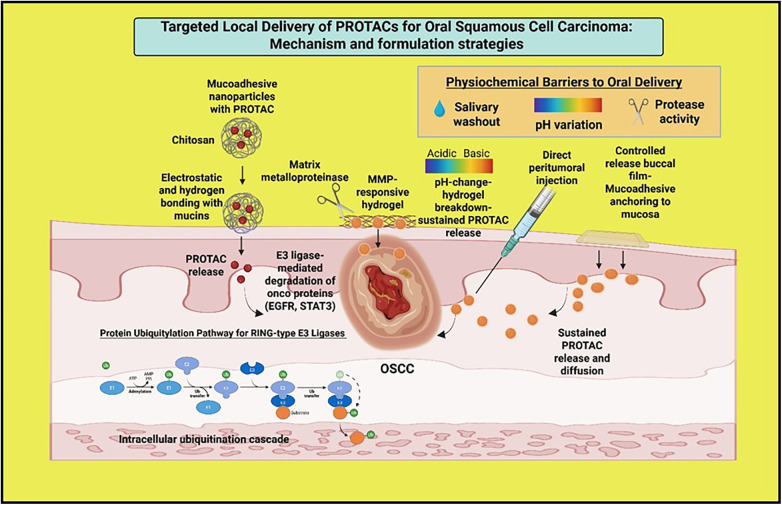
Targeted local delivery of PROTACs for OSCC. The schematic shows localized PROTAC delivery in OSCC via nanoparticles, buccal films, hydrogels, and injections, overcoming oral barriers and enabling diffusion, uptake, and E3 ligase-driven oncogenic protein degradation. Created in BioRender. Shamsi, M. (2025) https://BioRender.com/csctuq8.

Localized injection of PROTAC formulations into the perilesional tissue or surgical margins can provide therapeutic concentration at the tumor interface, which is crucial in OSCC, where recurrence often initiates at resection borders. This strategy reduces systemic toxicity and allows repeated dosing. Enzyme-responsive carriers that release PROTACs in response to matrix metalloproteinases or cathepsin enzymes upregulated in OSCC, are being evaluated in solid tumor models and offer relevance for oral cavity applications (24, 47). These advances indicate that delivery science tailored to the oral microenvironment can enhance the clinical viability of PROTACs for OSCC. Integration of formulation science with molecular oncology will be necessary to translate laboratory findings into topical or localized therapeutics for high-risk oral cancer. Figure 4 shows the localized PROTAC delivery in OSCC via nanoparticles, buccal films, hydrogels, and injections, overcoming oral barriers and enabling diffusion, uptake, and E3 ligase–driven oncogenic protein degradation.

## Natural product-based PROTACS

Natural products provide a foundation for anticancer therapeutics, and many phytochemicals show cytotoxic, antioxidant, or chemopreventive effects in OSCC. Incorporating these agents into PROTAC design offers a novel therapeutic direction. Several plant-derived bioactives, including flavonoids, terpenoids, and alkaloids, display target-binding activity and can function as PROTAC warheads (15, 53). It has been demonstrated in many studies that flavonoids such as quercetin, luteolin, apigenin, and epigallocatechin gallate (EGCG) inhibit oncogenic regulators, including EGFR, PI3K, STAT3, and c-MYC in OSCC models (16, 18, 51, 57). Their moderate affinity for protein targets, together with favorable safety, supports their role as potential scaffolds for PROTACs. Flavonoid interactions with bromodomain proteins such as BRD4, a driver of transcriptional reprogramming and chemoresistance in oral epithelial cancers (61). These interactions highlight phytochemical-inspired PROTACs as promising candidates for OSCC therapy. Alkaloids such as berberine and harmine target cyclin-dependent kinases (CDKs) and transcription factors, showing inhibition of CDK2- and MYC-associated pathways in head and neck cancer models (22, 58). Their planar scaffolds and DNA-binding motifs enhance nuclear permeability, an essential property for degrading intranuclear regulators like STAT3 and MYC. Incorporating these alkaloids into bifunctional PROTACs allows precise target engagement and proteasomal elimination. Plant-derived ligands contribute diverse pharmacophores that occupy shallow pockets or protein–protein interaction domains typically inaccessible to small-molecule inhibitors (62). It has been shown that natural products as starting points for warhead optimization, with PROTACs employing flavonoid warheads achieving BRD4 and HDAC degradation in solid tumor models. Many phytochemicals also modulate oxidative stress and inflammation in oral tissues, offering multi-targeted benefits that complement degradation (63).

Localised delivery systems strengthen this strategy. Pandey et al. developed mucoadhesive nanoparticle formulations that improved retention and mucosal bioavailability of flavonoid-based compounds. PEGylated resveratrol-associated PROTACs achieved higher stability and reduced microbial metabolism in oral models (64, 65). Coumarin-based PROTACs have also been engineered for theranostic applications, enabling both degradation and imaging *in vivo*, as shown by Cheng et al. (56), Chen et al. (66) reported that these constructs validated oncogenic targets such as EGFR, c-MYC, and CDK6 in various solid tumors. Recent computational screening studies predict that plant-derived ligands may effectively recruit E3 ligases such as CRBN or von Hippel-Lindau (VHL), suggesting new opportunities for personalized OSCC therapy (67). Natural products have been widely explored for chemoprevention in OSCC, particularly in patients with early-stage disease and OPMDs. Curcumin, resveratrol, and sulforaphane have been reported to suppress tumor initiation by modulating key signaling pathways such as NF-κB, p53, EGFR, and apoptosis regulators, including BCL-2 and MCL-1 (36, 68). These phytochemicals offer favorable safety profiles and demonstrate target engagement, making them attractive candidates as warheads in PROTACs (65, 69). Recent studies have designed curcumin-based analogs that were successfully incorporated into PROTAC constructs to degrade transcriptional co-activators and epigenetic modulators, leveraging their intrinsic binding properties and low toxicity (70). Combining dietary chemopreventive agents with PROTAC technology improves the cellular uptake, stabilizes targeted degradation pathways, and reduces pro-inflammatory signaling. This facilitates reducing the therapeutic dose, which is an essential advantage for prolonged OSCC chemoprevention strategies (54, 65, 69).

Natural product-based PROTACs rely on precise structural design governed by structure–activity relationships (SAR). Optimizing the warhead, linker, and E3 ligase recruiter determines potency and selectivity in OSCC. For flavonoid-based warheads like quercetin and EGCG, the hydroxyl group position affects target binding to EGFR and STAT3, but reduces the metabolic stability (16, 18, 51, 57). Methylation or glycosylation of reactive hydroxyl groups improves the pharmacokinetics and maintains target affinity. The replacement of the unstable diketone in curcumin with a heterocyclic bioisostere strengthens the metabolic stability and prolongs the cellular half-life while preserving NF-*κ*B degradation (70). For nuclear targets such as c-MYC and BRD4, PEG-based flexible linkers have been shown to enhance ternary complex formation (61). In the context of oral tumors, naturally derived molecules like resveratrol offer both targeting advantages and additional antioxidant properties (71). These structure–activity insights are helping drive the development of OSCC-specific degraders with improved potency, selectivity, and drug-like characteristics suitable for therapeutic application.

## Future perspectives

Sequencing-based detection of variants in STAT3, EGFR, or c-MYC can inform the development of PROTACs optimized with tailored warheads and linker architectures (28, 35, 72). *In vivo* validation is increasingly conducted using patient-derived xenografts (PDX) and organoid models of OSCC. Artificial intelligence (AI) and machine learning are now integrated into *de novo* discovery, enabling prediction of ternary complex formation, assessment of linker dynamics, and modelling of pharmacokinetics. Algorithms trained on degradation datasets further accelerate scaffold optimization for ligase recruitment (61, 73). AI-driven SAR modeling and docking particularly enhance efforts against difficult targets such as BRD4 or CDK6. Combining PROTACs with immunotherapy, chemotherapy, or radiotherapy creates new strategies to bypass resistance mechanisms in OSCC. Targeted degradation of immune checkpoint regulators or tumor antigens directly enhances responses to PD-1/PD-L1 blockade within immunosuppressive microenvironments (28, 34). PROTACs also sensitize tumor cells to radiation or platinum-based chemotherapy by degrading DNA repair proteins or apoptosis inhibitors such as MCL-1 and BCL-2 (12, 22). Preclinical studies currently evaluate sequential and concurrent protocols to define optimal dosing schedules. Theranostic approaches extend PROTAC utility by integrating imaging probes for real-time visualization of degradation kinetics, tumor penetration, and biodistribution. Dual-function agents, including coumarin-labelled and PET-traced PROTACs, validated protein degradation *in vivo* and linked it to therapeutic outcomes (56). These tools address tumor heterogeneity and field cancerization in OSCC by enabling personalized treatment through site-specific degradation monitoring and dosage adjustment. Translational goals now focus on oral-selective PROTACs engineered for the anatomical and immunological features of the oral cavity. Ligase profiling, microbiome integration, tissue-specific ligases, and modified linker chemistries improve therapeutic selectivity. Mucoadhesive, pH-sensitive delivery systems applied to dysplastic or recurrent lesions further enhance local efficacy while minimizing systemic toxicity.

## Conclusion

PROTACs provide a focused way to overcome major therapeutic limitations in OSCC. Their catalytic degradation mechanism removes difficult-to-target proteins, including transcription factors and anti-apoptotic regulators, which are essential for tumor growth. PROTACs, combined with molecular imaging and targeted delivery systems, can help in the diagnosis, treatment, and monitoring of diseases. Researchers are now concentrating on oral tissue–specific ligases and developing degraders which are customized according to patient profiles. Thus, the translation of these agents into clinical use requires close collaboration of chemists, molecular biologists, and oncologists. The use of PROTACs in oral cancer treatment could bring precise, patient-tailored therapy with longer-lasting results and improved clinical accuracy. While PROTAC strategies hold promise for OSCC therapy, further mechanistic and clinical studies are needed to validate their targets and establish their prognostic and therapeutic significance.

## References

[1] Ernani V Saba NF. Oral cavity cancer: risk factors, pathology, and management. Oncology. (2015) 89:187–95. 10.1159/000398801 26088938

[2] Chen S Yang X Huang L Xie Y Li Y Lin Y. Increasing incidence, prevalence, and mortality of lip and oral cavity cancer in adults aged 70 and older globally: findings from GBD 2021. World J Surg Oncol. (2025) 23:276. 10.1186/s12957-025-03933-9 40652241 PMC12255997

[3] Borse V Konwar AN Buragohain P. Oral cancer diagnosis and perspectives in India. Sens Int. (2020) 1:100046. 10.1016/j.sintl.2020.100046 34766046 PMC7515567

[4] Li H Dong J Cai M Xu Z Cheng XD Qin JJ. Protein degradation technology: a strategic paradigm shift in drug discovery. J Hematol Oncol. (2021) 14:138. 10.1186/s13045-021-01146-7 34488823 PMC8419833

[5] Mignogna MD Fedele S Lo Russo L. The world cancer report and the burden of oral cancer. Eur J Cancer Prev. (2004) 13:139–42. 10.1097/00008469-200404000-00008 15100581

[6] Kujan O Van Schaijik B Farah CS. Immune checkpoint inhibitors in oral cavity squamous cell carcinoma and oral potentially malignant disorders: a systematic review. Cancers (Basel). (2020) 12:1937. 10.3390/cancers12071937 32708945 PMC7409293

[7] Xie L Shang Z. Burden of oral cancer in Asia from 1990 to 2019: estimates from the global burden of disease 2019 study. PLoS One. (2022) 17:e0265950. 10.1371/journal.pone.0265950 35324990 PMC8947401

[8] Jiang M Li B. STAT3 and its targeting inhibitors in oral squamous cell carcinoma. Cells. (2022) 11:3131. 10.3390/cells11193131 36231093 PMC9563058

[9] Sen M Joyce S Panahandeh M Li C Thomas SM Maxwell J Targeting Stat3 abrogates EGFR inhibitor resistance in cancer. Clin Cancer Res. (2012) 18:4986–96. 10.1158/1078-0432.CCR-12-0792 22825581 PMC3445706

[10] Rahman QB Iocca O Kufta K Shanti RM. Global burden of head and neck cancer. Oral Maxillofac Surg Clin North Am. (2020) 32:367–75. 10.1016/j.coms.2020.04.002 32482563

[11] Lv M Hu W Zhang S He L Hu C Yang S. Proteolysis-targeting chimeras: a promising technique in cancer therapy for gaining insights into tumor development. Cancer Lett. (2022) 539:215716. 10.1016/j.canlet.2022.215716 35500825

[12] Shiah JV Grandis JR Johnson DE. Targeting STAT3 with proteolysis targeting chimeras and next-generation antisense oligonucleotides. Mol Cancer Ther. (2021) 20:219–28. 10.1158/1535-7163.MCT-20-0599 33203730 PMC7888537

[13] Morgan E Soerjomataram I Rumgay H Coleman HG Thrift AP Vignat J The global landscape of esophageal squamous cell carcinoma and esophageal adenocarcinoma incidence and mortality in 2020 and projections to 2040: new estimates from GLOBOCAN 2020. Gastroenterology. (2022) 163:649–e642. 10.1053/j.gastro.2022.05.054 35671803

[14] Rahadiani N Habiburrahman M Stephanie M Handjari DR Krisnuhoni E. Estimated projection of oral squamous cell carcinoma annual incidence from twenty years registry data: a retrospective cross-sectional study in Indonesia. PeerJ. (2023) 11:e15911. 10.7717/peerj.15911 37663292 PMC10473041

[15] Pei H Peng Y Zhao Q Chen Y. Small molecule PROTACs: an emerging technology for targeted therapy in drug discovery. RSC Adv. (2019) 9:16967–76. 10.1039/C9RA03423D 35519875 PMC9064693

[16] Liu Z Hu M Yang Y Du C Zhou H Liu C An overview of PROTACs: a promising drug discovery paradigm. Mol Biomed. (2022) 3:46. 10.1186/s43556-022-00112-0 36536188 PMC9763089

[17] Wu J Chen H Liu Y Yang R An N. The global, regional, and national burden of oral cancer, 1990–2021: a systematic analysis for the global burden of disease study 2021. J Cancer Res Clin Oncol. (2025) 151:53. 10.1007/s00432-025-06098-w 39875744 PMC11775039

[18] Zhang SZ Xie L Shang ZJ. Burden of oral cancer on the 10 most populous countries from 1990 to 2019: estimates from the global burden of disease study 2019. Int J Environ Res Public Health. (2022) 19;875. 10.3390/ijerph19020875 35055693 PMC8775770

[19] Qin L Dai H Wang J. Key considerations in targeted protein degradation drug discovery and development. Front Chem. (2022) 10:934337. 10.3389/fchem.2022.934337 35978859 PMC9376879

[20] Han X Sun Y. PROTACs: a novel strategy for cancer drug discovery and development. MedComm. (2023) 4:e290. 10.1002/mco2.290 37261210 PMC10227178

[21] Zhong G Chang X Xie W Zhou X. Targeted protein degradation: advances in drug discovery and clinical practice. Signal Transduct Target Ther. (2024) 9:308. 10.1038/s41392-024-02004-x 39500878 PMC11539257

[22] Kelm JM Pandey DS Malin E Kansou H Arora S Kumar R PROTAC'ing oncoproteins: targeted protein degradation for cancer therapy. Mol Cancer. (2023) 22:62. 10.1186/s12943-022-01707-5 36991452 PMC10061819

[23] Hanahan D Weinberg RA. Hallmarks of cancer: the next generation. Cell. (2011) 144:646–74. 10.1016/j.cell.2011.02.013 21376230

[24] Qi S-M Dong J Xu Z-Y Cheng X-D Zhang W-D Qin J-J. PROTAC: an effective targeted protein degradation strategy for cancer therapy. Front Pharmacol. (2021) 12:692574. 10.3389/fphar.2021.692574 34025443 PMC8138175

[25] Li X Pu W Zheng Q Ai M Chen S Peng Y. Proteolysis-targeting chimeras (PROTACs) in cancer therapy. Mol Cancer. (2022) 21:99. 10.1186/s12943-021-01434-3 35410300 PMC8996410

[26] Rahman QB Iocca O Kufta K Shanti RM. Global burden of head and neck cancer. Oral Maxillofac Surg Clin North Am. (2020) 32:367–75. 10.1016/j.coms.2020.04.002 32482563

[27] Shiah JV Grandis JR Johnson DE. Targeting STAT3 with proteolysis targeting chimeras and next-generation antisense oligonucleotides. Mol Cancer Ther. (2021) 20:219–28. 10.1158/1535-7163.MCT-20-0599 33203730 PMC7888537

[28] Tang J Chen H Fan H Chen T Pu C Guo Y. Research progress of BRD4 in head and neck squamous cell carcinoma: therapeutic application of novel strategies and mechanisms. Bioorg Med Chem. (2024) 113:117929. 10.1016/j.bmc.2024.117929 39317007

[29] Sasahira T Kirita T. Hallmarks of cancer-related newly prognostic factors of oral squamous cell carcinoma. Int J Mol Sci. (2018) 19:2413. 10.3390/ijms19082413 30115834 PMC6121568

[30] Mordzinska-Rak A Telejko I Adamczuk G Trombik T Stepulak A Blaszczak E. Advancing head and neck cancer therapies: from conventional treatments to emerging strategies. Biomedicines. (2025) 13:1046. 10.3390/biomedicines13051046 40426875 PMC12108569

[31] Jimenez DG Sebastiano J Caron G Ermondi G. Are we ready to design oral PROTACs®? ADMET and DMPK. (2021) 9:243–54. 35300370 10.5599/admet.1037 PMC8920102

[32] Qin L Dai H Wang J. Key considerations in targeted protein degradation drug discovery and development. Front Chem. (2022) 10:934337. 10.3389/fchem.2022.934337 35978859 PMC9376879

[33] Jiang H Xiong H Gu S-X Wang M. E3 ligase ligand optimization of clinical PROTACs. Front Chem. (2023) 11:1098331. 10.3389/fchem.2023.1098331 36733714 PMC9886873

[34] Jayaseelan VP Loganathan K Pandi A Ramasubramanian A Kannan B Arumugam P. Proteolysis-targeting chimeras targeting epigenetic modulators: a promising strategy for oral cancer therapy. Epigenomics. (2023) 15:1233–6. 10.2217/epi-2023-0293 37990892

[35] Nemec V Schwalm MP Muller S Knapp S. PROTAC degraders as chemical probes for studying target biology and target validation. Chem Soc Rev. (2022) 51:7971–93. 10.1039/D2CS00478J 36004812

[36] Leeman-Neill RJ Seethala RR Singh SV Freilino ML Bednash JS Thomas SM Inhibition of EGFR-STAT3 signaling with erlotinib prevents carcinogenesis in a chemically-induced mouse model of oral squamous cell carcinoma. Cancer Prev Res (Phila). (2011) 4:230–7. 10.1158/1940-6207.CAPR-10-0249 21163936 PMC3076320

[37] Mitic D Jaksic Karisik M Lazarevic M Carkic J Zivkovic E Mitrovic Ajtic O Oral squamous cell carcinoma exosomes upregulate PIK3/AKT, PTEN, and NOTCH signaling pathways in normal fibroblasts. Curr Issues Mol Biol. (2025) 47:568. 10.3390/cimb47070568 40729037 PMC12293655

[38] Wang C Zhang Y Chen W Wu Y Xing D. New-generation advanced PROTACs as potential therapeutic agents in cancer therapy. Mol Cancer. (2024) 23:110. 10.1186/s12943-024-02024-9 38773495 PMC11107062

[39] Vikal A Maurya R Patel BB Sharma R Patel P Patil UK Protacs in cancer therapy: mechanisms, design, clinical trials, and future directions. Drug Deliv Transl Res. (2025) 15:1801–27. 10.1007/s13346-024-01754-z 39614036

[40] Jin J Wu Y Zhao Z Wu Y Zhou Y-D Liu S Small-molecule PROTAC mediates targeted protein degradation to treat STAT3-dependent epithelial cancer. JCI insight. (2022) 7:e160606. 10.1172/jci.insight.160606 36509291 PMC9746828

[41] Cieslak M Slowianek M. Cereblon-recruiting PROTACs: will new drugs have to face old challenges? Pharmaceutics. (2023) 15:812. 10.3390/pharmaceutics15030812 36986673 PMC10053963

[42] Yedla P Babalghith AO Andra VV Syed R. PROTACs in the management of prostate cancer. Molecules. (2023) 28:3698. 10.3390/molecules28093698 37175108 PMC10179857

[43] Wang J Rong Q Ye L Fang B Zhao Y Sun Y Discovery of a novel orally bioavailable FLT3-PROTAC degrader for efficient treatment of acute myeloid leukemia and overcoming resistance of FLT3 inhibitors. J Med Chem. (2024) 67:7197–223. 10.1021/acs.jmedchem.4c00051 38655686

[44] Bekes M Langley DR Crews CM. PROTAC targeted protein degraders: the past is prologue. Nat Rev Drug Discov. (2022) 21:181–200. 10.1038/s41573-021-00371-6 35042991 PMC8765495

[45] Jung H Lee Y. Targeting the undruggable: recent progress in PROTAC-induced transcription factor degradation. Cancers (Basel). (2025) 17:1871 10.3390/cancers17111871 40507351 PMC12153801

[46] Xiong J Li G Mei X Ding J Shen H Zhu D Co-delivery of p53 restored and E7 targeted nucleic acids by poly (Beta-amino ester) complex nanoparticles for the treatment of HPV related cervical lesions. Front Pharmacol. (2022) 13:826771. 10.3389/fphar.2022.826771 35185576 PMC8855959

[47] Apprato G Poongavanam V Garcia Jimenez D Atilaw Y Erdelyi M Ermondi G Exploring the chemical space of orally bioavailable PROTACs. Drug Discov Today. (2024) 29:103917. 10.1016/j.drudis.2024.103917 38360147

[48] Mullard A. Arvinas’s PROTACs pass first safety and PK analysis. Nat Rev Drug Discov. (2019) 18:895. 10.1038/d41573-019-00188-4 31780851

[49] Wang X Xin L Deng X Dong C Hu G Zhou HB. Fluorescence theranostic PROTACs for real-time visualization of ERalpha degradation. Eur J Med Chem. (2024) 267:116184. 10.1016/j.ejmech.2024.116184 38320426

[50] Schade M Scott JS Hayhow TG Pike A Terstiege I Ahlqvist M Structural and physicochemical features of oral PROTACs. J Med Chem. (2024) 67:13106–16. 10.1021/acs.jmedchem.4c01017 39078401

[51] Sun G Rong D Li Z Sun G Wu F Li X Role of small molecule targeted compounds in cancer: progress, opportunities, and challenges. Front Cell Dev Biol. (2021) 9:694363. 10.3389/fcell.2021.694363 34568317 PMC8455877

[52] Nieto-Jiménez C Morafraile EC Alonso-Moreno C Ocaña A. Clinical considerations for the design of PROTACs in cancer. Mol Cancer. (2022) 21:67. 10.1186/s12943-022-01535-7 35249548 PMC8900451

[53] Han X Sun Y. Strategies for the discovery of oral PROTAC degraders aimed at cancer therapy. Cell Rep Physic Sci. (2022) 3:101062. 10.1016/j.xcrp.2022.101062

[54] Kamaraj R Ghosh S Das S Sen S Kumar P Majumdar M Targeted protein degradation (TPD) for immunotherapy: understanding proteolysis targeting chimera-driven ubiquitin-proteasome interactions. Bioconjugate Chem. (2024) 35:1089–115. 10.1021/acs.bioconjchem.4c00253 PMC11342303 38990186

[55] Hinterndorfer M Spiteri VA Ciulli A Winter GE. Targeted protein degradation for cancer therapy. Nat Rev Cancer. (2025) 25:493–516. 10.1038/s41568-025-00817-8 40281114

[56] Cheng J He S Xu J Huang M Dong G Sheng C. Making protein degradation visible: discovery of theranostic PROTACs for detecting and degrading NAMPT. J Med Chem. (2022) 65:15725–37. 10.1021/acs.jmedchem.2c01243 36442664

[57] Liu Z Zhang Y Xiang Y Kang X. Small-molecule PROTACs for cancer immunotherapy. Molecules. (2022) 27:5439. 10.3390/molecules27175439 36080223 PMC9458232

[58] Li T Zong Q Dong H Ullah I Pan Z Yuan Y. Non-invasive *in vivo* monitoring of PROTAC-mediated protein degradation using an environment-sensitive reporter. Nat Commun. (2025) 16:1892. 10.1038/s41467-025-57191-0 39987172 PMC11846984

[59] Liu X Wang A Shi Y Dai M Liu M Cai HB. PROTACs in epigenetic cancer therapy: current status and future opportunities. Molecules. (2023) 28:1217. 10.3390/molecules28031217 36770884 PMC9919707

[60] Juan A Del Mar Noblejas-Lopez M Arenas-Moreira M Alonso-Moreno C Ocana A. Options to improve the action of PROTACs in cancer: development of controlled delivery nanoparticles. Front Cell Dev Biol. (2021) 9:805336. 10.3389/fcell.2021.805336 35186955 PMC8851355

[61] Yamamoto T Hirosue A Nakamoto M Yoshida R Sakata J Matsuoka Y BRD4 promotes metastatic potential in oral squamous cell carcinoma through the epigenetic regulation of the MMP2 gene. Br J Cancer. (2020) 123:580–90. 10.1038/s41416-020-0907-6 32499570 PMC7435185

[62] Luo H Tian Y Abdullah R Zhang B Ma Y Zhang G. Advancing design strategy of PROTACs for cancer therapy. MedComm. (2025) 6:e70258. 10.1002/mco2.70258 40567248 PMC12188103

[63] Li Y Jia Y Wang X Shang H Tian Y. Protein-targeted degradation agents based on natural products. Pharmaceuticals (Basel). (2022) 16:46. 10.3390/ph16010046 36678543 PMC9865760

[64] Pandey M Choudhury H Ying JNS Ling JFS Ting J Ting JSS Mucoadhesive nanocarriers as a promising strategy to enhance intracellular delivery against oral cavity carcinoma. Pharmaceutics. (2022) 14:795. 10.3390/pharmaceutics14040795 35456629 PMC9025168

[65] Sflakidou E Leonidis G Foroglou E Siokatas C Sarli V. Recent advances in natural product-based hybrids as anti-cancer agents. Molecules. (2022) 27:6632. 10.3390/molecules27196632 36235168 PMC9572494

[66] Chen Y Yang Q Xu J Tang L Zhang Y Du F PROTACs in gastrointestinal cancers. Mol Ther Oncolytics. (2022) 27:204–23. 10.1016/j.omto.2022.10.012 36420306 PMC9676279

[67] Girardini M Maniaci C Hughes SJ Testa A Ciulli A. Cereblon versus VHL: hijacking E3 ligases against each other using PROTACs. Bioorg Med Chem. (2019) 27:2466–79. 10.1016/j.bmc.2019.02.048 30826187 PMC6561380

[68] Ma L Kim MO. Advances in preventive and therapeutic strategies for oral cancer: a short review. J Cancer Prev. (2024) 29:113–9. 10.15430/JCP.24.027 39790224 PMC11706729

[69] Al-Awadhi FH Luesch H. Targeting eukaryotic proteases for natural products-based drug development. Nat Prod Rep. (2020) 37:827–60. 10.1039/C9NP00060G 32519686 PMC7406119

[70] Zhang Z Xu M Shi R He X Wang Y Shao Y Natural compound-rhein and PROTACs unleash potent VEGFR-2 degraders. Chem Biodivers. (2024) 21:e202400753. 10.1002/cbdv.202400753 38818648

[71] Gough SM Flanagan JJ Teh J Andreoli M Rousseau E Pannone M Oral estrogen receptor PROTAC vepdegestrant (ARV-471) is highly efficacious as monotherapy and in combination with CDK4/6 or PI3K/mTOR pathway inhibitors in preclinical ER+ breast cancer models. Clin Cancer Res. (2024) 30:3549–63. 10.1158/1078-0432.CCR-23-3465 38819400 PMC11325148

[72] Omar EA Rajesh R Das PK Pal R Purawarga Matada GS Maji L. Next-generation cancer therapeutics: PROTACs and the role of heterocyclic warheads in targeting resistance. Eur J Med Chem. (2025) 281:117034. 10.1016/j.ejmech.2024.117034 39527893

[73] Tan S Chen Z Lu R Liu H Yao X. Rational proteolysis targeting chimera design driven by molecular modeling and machine learning. Wiley Interdiscip Rev Comput Mol Sci. (2025) 15(2):e70013.

[74] Sa JO Trino LD Oliveira AK Lopes AFB Granato DC Normando AGC Proteomic approaches to assist in diagnosis and prognosis of oral cancer. Expert Rev Proteomics. (2021) 18:261–84. 10.1080/14789450.2021.1924685 33945368

[75] Yao Y Wu M Wang Y Liao Z Yang Y Liu Y An oral PROTAC targeting HPK1 degradation potentiates anti-solid tumor immunity. Adv Mater. (2025) 37:e2411454. 10.1002/adma.202411454 39568237

[76] Zhong L Li Y Xiong L Wang W Wu M Yuan T Small molecules in targeted cancer therapy: advances, challenges, and future perspectives. Signal Transduct Target Ther. (2021) 6:201. 10.1038/s41392-021-00572-w 34054126 PMC8165101

